# Natural Killer Cell Activity and Interleukin-12 in Metabolically Healthy versus Metabolically Unhealthy Overweight Individuals

**DOI:** 10.3389/fimmu.2017.01700

**Published:** 2017-11-29

**Authors:** Minjoo Kim, Minkyung Kim, Hye Jin Yoo, Jong Ho Lee

**Affiliations:** ^1^Research Center for Silver Science, Institute of Symbiotic Life-TECH, Yonsei University, Seoul, South Korea; ^2^Department of Food and Nutrition, Brain Korea 21 PLUS Project, College of Human Ecology, Yonsei University, Seoul, South Korea; ^3^National Leading Research Laboratory of Clinical Nutrigenetics/Nutrigenomics, Department of Food and Nutrition, College of Human Ecology, Yonsei University, Seoul, South Korea

**Keywords:** metabolically healthy overweight, metabolically unhealthy overweight, natural killer cell, interleukin-12, immune system

## Abstract

The purpose of this study was to determine whether the immune system is involved in the different metabolic circumstances in healthy and unhealthy overweight individuals. We examined the metabolic and immune characteristics of 117 overweight individuals. Subjects were classified as metabolically healthy overweight (MHO, *n* = 72) or metabolically unhealthy overweight (MUO, *n* = 45). The immune response was measured by circulating levels of natural killer (NK) cell activity and cytokines. Both groups were comparable with regards to age, sex distribution, smoking and drinking status, and body mass index. When compared to the MHO group, the MUO group showed higher systolic and diastolic blood pressure, serum levels of triglyceride, glucose, glucose-related markers, and lower levels of HDL cholesterol. Compared to the MHO group, the MUO group showed 39% lower interferon-γ levels (not significant) and 41% lower interleukin (IL)-12 levels (significant). The MUO group also showed lower NK cell activity at E:T ratios of 10:1, 5:1, 2.5:1, and 1.25:1 (all *P*s < 0.05) than the MHO group. This study indicates that individuals displaying the MUO phenotype present an unfavorable immune system with lower NK cell activities under all assay conditions and lower serum levels of IL-12 than the activities and levels in similarly overweight MHO individuals. This result suggests that the immune system may be altered in overweight individuals who are at risk for overweight/obesity-related comorbidities.

## Introduction

The prevalence of overweight and obesity, which are defined as excessive fat accumulation, has increased rapidly worldwide. More than half (53.8%) of the adult in The Organization for Economic Co-operation and Development countries are overweight or obese (1). Even societies which consist by lean subjects are beginning to experience the impacts associated with an increasing prevalence of obesity. The prevalence of overweight/obesity in Korea consistently increase nearly 33.2% of adults over the age of 20 (2).

An increased body mass index (BMI) is crucial for up to 30% of various cancers, particularly pancreatic, colorectal cancer, etc. (3, 4), and it has recently been reported that the cancer risks caused by an increased BMI are remediable (5, 6). A subgroup of obese individuals is protected against the development of insulin resistance (IR), chronic inflammation, and metabolic abnormalities associated with obesity, and these individuals are classified as obese but metabolically healthy (7). Depending on the definition of metabolically healthy, 9–41% of obese individuals are classified as metabolically healthy obese (8, 9). Nevertheless, the etiologies underlying the heterogeneous metabolic phenotypes in those who are metabolically healthy overweight (MHO) or metabolically unhealthy overweight (MUO) are poorly understood (10).

The immunometabolic interaction in overweight/obese individuals is currently considered a key factor in chronic low-grade inflammation (11). For example, in diabetic obese mice, metastasis significantly promoted reduction of natural killer (NK) cell function (12). Additionally, compared to lean mice, diet-induced obese mice have decreased NK cytotoxicity and showed high mortality rates (13). A recent study showed decreased immune function in human NK cells after long-term exposure to leptin, which is produced by adipocytes and is the product of the obese gene (14). Furthermore, reduced expression of tumor necrosis factor (TNF)-related apoptosis-inducing ligand and p-Janus family tyrosine kinase 2 in NK cells was considered one mechanism by which chronically increased leptin levels cause NK cell dysfunctions in obese individuals (15). Thus, NK cells are crucial for the body’s defense against infection and in tumor surveillance. However, the link between different overweight/obese phenotypes and immune status remains unclear. Therefore, our purpose is to determine whether the immune system is involved in the different metabolic circumstances of healthy and unhealthy overweight individuals.

## Materials and Methods

### Subjects

Study subjects were recruited through advertisements by the Clinical Nutrigenetics/Nutrigenomics Laboratory at Yonsei University from March 2016 to February 2017. Volunteers who agreed to participate were screened to measure BMI and personal history of any diseases. After screening, subjects who were overweight or obese (BMI ≥ 25 kg/m^2^, World Health Organization definitions) and aged 40–69 years were enrolled. The exclusion criteria included cardiovascular disease, cancer, immune disease, liver disease, renal disease, pregnancy, and regular dietary supplement use. All participants provided written informed consent and the Institutional Review Board of Yonsei University approved the study protocol, which complied with the Declaration of Helsinki. Sample size was determined and calculated using R software v.3.4.1 with package “pwr.” In an exploratory pilot study, the NK cell activity at an E:T ratio of 10:1 in the MUO group was 20.57 ± 19.74% (mean ± standard deviation) lower than that in the MHO group (31.69 ± 18.08%). The sample size was determined *via* a two-sample *t*-test power calculation with effect size (*d* = 0.596), power of 0.8, and level of significance (α = 0.05). The result indicated that a minimum of 45 subjects per group were needed, and thus, we selected an MUO:MHO ratio of 1:1.6 to increase statistical power for the test.

### Definition of MHO and MUO Groups

Participants were classified as MHO (*n* = 72) or MUO (*n* = 45) based on the definition of the National Cholesterol Education Program Expert Panel on Detection, Evaluation, and Treatment of High Blood Cholesterol in Adults (Adult Treatment Panel III). In detail, the definition of “metabolically unhealthy” was described in previous published study (16) as the presence of three or more of the five criteria.

### Anthropometry, Dietary Intake, and Sample Collection

All anthropometry information is previously described (16). To calculate BMI (kilograms per square meter), weight and height were measured. Waist circumstances and BPs were assessed using automatic BP monitor. The 3-day diet record was obtained and the amount of diet intake was calculated using nutritional analysis software (Data S1 in Presentation S1 in Supplementary Material). After overnight fast of at least 12 h, venous blood samples were collected in EDTA-treated tubes and serum tubes. After centrifugation, plasma and serum samples were stored at −80°C.

### Laboratory Assessments

To assess the lipid profile, serum TG, total cholesterol, and HDL cholesterol were measured using kits and auto analyzer and LDL cholesterol was calculated by the Friedewald formula. Fasting serum glucose and glucose-related markers are also measured including insulin and homeostatic model assessment of IR (HOMA-IR) index. These laboratory assessments including lipid profile and glucose-related markers are previously described in detail (16). A Hitachi 7600 autoanalyzer (Hitachi, Tokyo, Japan) was used to measure the following: serum alanine aminotransferase (ALT) levels *via* the International Federation of Clinical Chemistry—Ultraviolet method, serum gamma-glutamyltransferase (GGT) levels *via* a modified Sanz method, blood urea nitrogen (BUN) *via* a kinetic UV assay for urea/urea nitrogen, creatinine levels *via* the creatinine Jaffe method, serum albumin concentrations *via* the bromocresol green method, and serum high-sensitivity C-reactive protein (hs-CRP) levels *via* a latex-agglutination turbidimetric immunoassay. Plasma adiponectin concentrations were measured by an enzyme immunoassay (Data S2 in Presentation S2 in Supplementary Material). The serum level of cytokines including interleukin (IL)-6, IL-1β, TNF-α, interferon (IFN)-γ, and IL-12 were measured using kits (Data S2 in Presentation S2 in Supplementary Material).

### Isolation of Peripheral Blood Mononuclear Cells (PBMCs)

Detailed descriptions are provided in previous published study (17) and described in Data S3 in Presentation S3 in Supplementary Material. Whole blood was mixed with RPMI 1640 medium (ratio of 1:1) and Histopaque-1077 gradient was used for separation. Isolated PBMCs were cultured with RPMI 1640 containing penicillin/streptomycin (Pen/Strep) for NK cell cytotoxicity assays.

### Determination of NK Cell Activity

Detailed descriptions are provided in previous published study (17) described in Data S4 in Presentation S4 in Supplementary Material. To assay NK cell cytotoxic activity, isolated PBMCs (effector cells, E), which firstly incubated with K-562 cells, were seeded with 2 × 10^4^ K-562 cells (target cells, T) with the four serial dilutions (E:T ratios of 10:1, 5:1, 2.5:1, and 1.25:1). The following formula was used to obtain% cytotoxicity for respective E;T ratio:

$$\text{\% cytotoxicity}=\frac{\text{experimental}–\text{effector spontaneous}–\text{target spontaneous}}{\text{target maximum}–\text{target spontaneous}}\times 100.$$

### Statistical Analysis

SPSS statistics 24.0 software (IBM, Chicago, IL, USA) was used for overall test; independent *t*-test, chi-square test, Pearson’s correlation coefficient. Skewed variables were log transformed. Overall statistical analysis was reported at significant at two-tailed *P* value < 0.05.

## Results

### Characteristics of MHO and MUO Groups

Table 1 shows the clinical and biochemical characteristics of the MHO and MUO groups. Both groups were comparable with regards to age, sex distribution, smoking and drinking status, and BMI. Compared with the MHO group, the MUO group showed higher SBP, DBP, and serum levels of TG, glucose, insulin, and HOMA-IR index and lower levels of HDL cholesterol. The MUO group also showed higher levels of ALT, GGT, and hs-CRP and lower concentrations of adiponectin than the MHO group (Table 1). Furthermore, although there were small differences in energy intake (26.7 kcal), as well as in three major nutrients, these differences were not statistically significant between the two groups (data not shown).

**Table 1:** Clinical and biochemical characteristics of the study population according to their metabolic and BMI status.

	MHO (*n* = 72)	MUO (*n* = 45)	*P*
Age (year)	56.2 ± 1.02	57.3 ± 1.35	0.529
Male/female, *n* (%)	16 (22.2)/56 (77.8)	9 (20.0)/36 (80.0)	0.775
Current smoker, *n* (%)	6 (8.3)	3 (6.7)	0.742
Current drinker, *n* (%)	44 (61.1)	25 (55.6)	0.552
BMI (kg/m^2^)	27.4 ± 0.17	27.6 ± 0.33	0.532
Waist (cm)	89.9 ± 0.74	92.2 ± 1.08	0.071
SBP (mmHg)	119.1 ± 1.53	132.6 ± 2.16	**<0.001**
DBP (mmHg)	75.6 ± 1.08	84.0 ± 1.34	**<0.001**
TG (mg/dL)^a^	106.6 ± 5.08	167.1 ± 11.6	**<0.001**
Total cholesterol (mg/dL)^a^	209.9 ± 3.87	213.6 ± 6.32	0.802
HDL cholesterol (mg/dL)^a^	56.2 ± 1.31	47.0 ± 1.59	**<0.001**
LDL cholesterol (mg/dL)^a^	132.4 ± 3.78	134.0 ± 5.45	0.963
Glucose (mg/dL)^a^	89.1 ± 0.96	96.3 ± 2.10	**0.003**
Insulin (μIU/dL)^a^	11.5 ± 0.47	13.7 ± 0.68	**0.002**
HOMA-IR^a^	2.53 ± 0.11	3.24 ± 0.16	**<0.001**
ALT (IU/L)^a^	19.0 ± 1.16	25.0 ± 2.25	**0.007**
GGT (IU/L)^a^	20.2 ± 1.26	27.8 ± 2.42	**0.004**
Albumin (mg/dL)^a^	4.64 ± 0.02	4.69 ± 0.04	0.284
BUN (mg/dL)^a^	14.2 ± 0.37	14.1 ± 0.53	0.740
Creatinine (mg/dL)^a^	0.71 ± 0.02	0.71 ± 0.03	0.853
hs-CRP (mg/dL)^a^	0.88 ± 0.09	1.68 ± 0.29	**0.035**
Adiponectin (ng/mL)^a^	8.22 ± 1.40	4.43 ± 0.59	**0.032**

*Mean ± SE. P values derived from an independent t-test*.

*P values below 0.05 are highlighted in boldface*.

*^a^Tested by logarithmic transformation*.

### Circulating Levels of Cytokines and NK Cell Activity

Table 2 shows the circulating levels of cytokines and NK cell activity. There were no significant differences in the levels of TNF-α, IL-1β, or IL-6 between the MUO and MHO groups. However, compared to the MHO group, the MUO group showed 39% lower IFN-γ levels (not significant) and 41% lower IL-12 levels (significant). The MUO group showed lower NK cell activity at an E:T ratio of 10:1 (*P* = 0.013), 5:1 (*P* = 0.043), 2.5:1 (*P* = 0.040), and 1.25:1 (*P* = 0.012) than the MHO group (Table 2). Furthermore, similar significant differences in immunological outcomes were found in male and female subjects (data not shown).

**Table 2:** Clinical and biochemical characteristics of the MHO and MUO groups.

	MHO (*n* = 72)	MUO (*n* = 45)	*P*
TNF-α (pg/mL)^a^	14.1 ± 1.83	12.3 ± 1.77	0.355
IL-1β (pg/mL)^a^	1.08 ± 0.16	0.80 ± 0.12	0.542
IL-6 (pg/mL)^a^	5.26 ± 0.67	5.37 ± 0.96	0.521
IL-12 (pg/mL)^a^	43.8 ± 7.45	26.0 ± 3.40	**0.030**
IFN-γ (pg/mL)^a^	16.6 ± 2.93	10.1 ± 1.49	0.625
NK cell activity 10:1 (%)	29.2 ± 2.61	20.5 ± 2.07	**0.013**
NK cell activity 5:1 (%)^a^	18.5 ± 1.63	12.2 ± 1.94	**0.043**
NK cell activity 2.5:1 (%)^a^	17.4 ± 2.15	7.78 ± 1.88	**0.040**
NK cell activity 1.25:1 (%)^a^	15.7 ± 1.84	7.52 ± 1.88	**0.012**

*Mean ± SE. P values derived from an independent t-test*.

*P values below 0.05 are highlighted in boldface*.

*^a^Tested by logarithmic transformation*.

### Correlations between NK Cell Activity and Cytokines

There was a positive correlation between IFN-γ and NK cell activity at an E:T ratio of 2.5:1 (*r* = 0.334, *P* = 0.002), and positive correlation trends were shown between IFN-γ levels and NK cell activity at an E:T ratio of 5:1 (*r* = 0.179, *P* = 0.095). Although a positive correlation was also shown between IFN-γ and IL-12 levels (*r* = 0.241, *P* = 0.023), our results did not show any correlation between IL-12 and NK cell activity at any E:T ratio.

## Discussion

Overweight/obese populations are not a homogenous group. In accordance with the Third National Health and Nutrition Examination Survey criteria, metabolic syndrome (MS) increases with age, and estimates indicate that in the age category of over 50 years, MS influences more than 40 and 30% of the population in US and Europe, respectively (18–20). When overweight subjects in this study were classified as either metabolically healthy or unhealthy, MUO individuals represented 38% of the total subjects. Compared to the MUO individuals in this study, the MHO individuals demonstrated more favorable mean levels of BP, glucose, and lipid profiles despite similar BMI to the MUO group. However, the factor that preserves this group from the expected results of excessive adiposity remains unclear. We focused on the immune system within the MHO and MUO groups and showed that the MUO group had lower serum NK cell activity, at overall E:T ratios, than the MHO group. This result expands a previous finding of significantly higher levels of NK cells and cytotoxic T lymphocytes in a metabolically healthy obese group than in a metabolically unhealthy obese group (21).

Natural killer cells represent a key element of innate immunity, as these cells secrete different cytokines (for example, IFN-γ) to stimulate other immune cells and can directly destroy virus-infected or malignant-transformed cells (22, 23). In the present study, compared to the MHO group, the MUO group showed 39% lower IFN-γ levels (not significant) and 41% lower IL-12 levels (significant). IL-12 plays a major role to induce IFN-γ released by NK cells and increases NK cell cytotoxicity (24). NK cells express various receptors on surface for recognizing and binding diverse targets (25, 26). Indeed, the results of an over 10-year longitudinal study showed an association between lower NK cell activity and an elevated risk of cancer development (27). Unlike the differences in NK cell activity and IL-12 levels, there were no significant differences in the levels of TNF-α, IL-1β, or IL-6 between the MUO and MHO groups. This result suggests that NK cell activity and IL-12 levels could be better indicators for predicting individuals who are at early immune risk than serum levels of TNF-α, IL-1β, and IL-6.

Interleukin-12 induces increased proliferation; enhanced cytotoxicity and expression of cytotoxic mediators; and increased production of cytokines, especially IFN-γ, in NK cells, T cells, and natural killer T (NKT) cells (28). However, although also mediated by T cells, IL-12-induced IFN-γ production is mainly mediated by NK cells (29, 30). Indeed, in our study, the levels of IFN-γ and IL-12 were positively correlated (*r* = 0.241, *P* = 0.023), but there was no relevant correlation between IL-12 levels and NK cell activity. Although there was no direct correlation between NK cell activity and IFN-γ or NK cell activity and IL-12 in the present study, the levels of IFN-γ, IL-12, and NK cell activity were lower in the MUO group, and this effect might be due to IL-12-induced IFN-γ production mediated by T cells. We could not prove the exact mechanism involved in the reduced levels of NK cell activity and IL-12 in the MUO group; nevertheless, our results suggested that the immune system may be altered in overweight subjects with unfavorable metabolic phenotype.

The NK subpopulation can be phenotypically defined using several surface antigens such as cluster of differentiation (CD)56, CD16, and CD3 and by expressing different degrees of lytic activity (21, 31). Peripheral blood NK cells are composed of different cell subsets and NK cells are generally identified by CD56. (CD^3−^)CD56^bright^ cells were mainly considered to be cytokine producers, whereas (CD^3−^)CD56^dim^ cells were considered to be cytotoxic effector cells (32). Upon stimulation with IL-12 or IL-2, CD56^bright^ NK cells produce IFN-γ (33). CD56^dim^CD16^bright^ cells represent the large majority of peripheral blood NK cells (32, 34), of which the expression of CD16—a low affinity Fc receptor—inversely correlated with CD56 expression in humans. Laue et al. (15) reported significantly lower levels of NKT cells and NK cell functional marker in obese subjects, which supports the impaired protective activity caused by obesity. Unhealthy obese patients, compared with counterparts, have markedly more NK cells that express the markers which inhibits cytolytic functions of NK cells including CD158b and NKB1 (21). Although the present study did not include information on the NK cell phenotype, we could speculate that, in addition to altered overall NK cell activities in MUO group, the NK cell phenotype might differ between the two groups.

Excessive body fat mass elevates circulating adipokine levels, which affect immune responses and functions (35, 36). NK cell functionality can be modulated by adipokines (37, 38). Previous studies have shown impaired NK cell functions regarding the leptin-signaling pathway in diet-induced obese animal models, furthermore, a potential reversible defects in NK cell function after weight loss (39, 40). Moulin et al. (41) showed that the reduced body weight causes decreased plasma leptin levels and enhanced NK cell activity and IFN-γ production after bariatric surgery. Leptin can antagonize the actions of adiponectin (42). Adiponectin might have anticancer effects due to its anti-inflammatory characteristic and is a negative regulator of angiogenesis (43). In this study, compared to the MHO group, the MUO group showed significantly lower adiponectin and higher hs-CRP levels, which were associated with an augmented risk of developing many types of cancers.

Chronic inhalation of cigarette smoke changes immunological responses (44). The influences of cigarette smoke over the immune system are dependent on the duration of smoking and individual’s sex. Several researches have studied the impacts of cigarette smoking on the NK cell function due to their influence on increased risk of various cancer. NK cell activity against cancer cells was markedly decreased in smokers compared to non-smokers (45–47). Furthermore, in mice model, resistance to the growth of tumor cell significantly decreases during cigarette smoke exposure time increases (48). These studies reflect that the increased risk of cancers in smokers might partially result from the impact of cigarette smoke on the immune system. In the present study, there were only six smokers and three smokers in the MHO and MUO groups, respectively (Table 1), with an absence of significant differences between two groups. We consider that having only a few smokers in this study may not affect the effect observed on the immune system; therefore, we did not include smoking status in the exclusion criteria.

Despite several intriguing findings that explain the differences between the MUO and MHO groups, the current study has limitations. First, although we matched for several potential confounding factors such as age, sex, and BMI, we had a moderate sample size. Second, we could not determine any causations between MS and abnormal system or other inflammatory risk factors through this cross-sectional study design. Third, the phenotype of NK cells, which express differing degrees of lytic activity, and markers of NK cell activation were not assessed in this study. Lastly, the underlying mechanism of the relationship between MS and an altered immune system was not clarified in this study. Individual components of MS are related risk factors for an abnormal immune system, but how the clustering of these components is connected with the development of immune dysfunction remains unknown. It seems obvious that a condition characterized by multiple risk factors, such as MS, resulted in a greater risk for adverse effects than a single risk factor.

In conclusion, the results of the current study indicate that individuals displaying the MUO phenotype present an unfavorable immune system with lower NK cell activities under all assay conditions and lower levels of IL-12 compared to similarly overweight MHO individuals. These lower levels of NK cell activity and IL-12 expression in MUO individuals suggest that the immune system may be altered in overweight population who are at risk for overweight/obesity-related comorbidities.

## Ethics Statement

The aim of this study was carefully explained to all participants, and each participant provided written informed consent. The Institutional Review Board of Yonsei University approved the study protocol, which complied with the Declaration of Helsinki.

## Author Contributions

MinkyungK and HY contributed to the acquisition, analysis, and interpretation of the data and helped draft the manuscript. MinjooK and JL contributed to the conception and design of the research and the analysis and interpretation of the data and drafted the manuscript. All authors critically revised the manuscript, read and approved the final manuscript, and agreed to be fully accountable for ensuring the integrity and accuracy of the work.

## Conflict of Interest Statement

The authors declare that the research was conducted in the absence of any commercial or financial relationships that could be construed as a potential conflict of interest.
