# Corrigendum: Natural Killer Cell Activity and Interleukin-12 in Metabolically Healthy versus Metabolically Unhealthy Overweight Individuals

**DOI:** 10.3389/fimmu.2018.02179

**Published:** 2018-09-20

**Authors:** Minjoo Kim, Minkyung Kim, Hye Jin Yoo, Jong Ho Lee

**Affiliations:** ^1^Research Center for Silver Science, Institute of Symbiotic Life-TECH, Yonsei University, Seoul, South Korea; ^2^Department of Food and Nutrition, Brain Korea 21 PLUS Project, College of Human Ecology, Yonsei University, Seoul, South Korea; ^3^National Leading Research Laboratory of Clinical Nutrigenetics/Nutrigenomics, Department of Food and Nutrition, College of Human Ecology, Yonsei University, Seoul, South Korea

**Keywords:** metabolically healthy overweight, metabolically unhealthy overweight, natural killer cell, interleukin-12, immune system

In the original article, there were typos with regard to the recruitment period and age of enrolled participants in MATERIALS AND METHODS, Subjects, 1st paragraph:

Study subjects were recruited through advertisements by the Clinical Nutrigenetics/Nutrigenomics Laboratory at Yonsei University from May 2014 to April 2017. Volunteers who agreed to participate were screened to measure BMI and personal history of any diseases. After screening, subjects who were overweight or obese (BMI ≥ 25 kg/m^2^, World Health Organization definitions) and aged 40–65 years were enrolled. The exclusion criteria included cardiovascular disease, cancer, immune disease, liver disease, renal disease, pregnancy, and regular dietary supplement use. All participants provided written informed consent and the Institutional Review Board of Yonsei University approved the study protocol, which complied with the Declaration of Helsinki. Sample size was determined and calculated using R software v.3.4.1 with package “pwr.” In an exploratory pilot study, the NK cell activity at an E:T ratio of 10:1 in the MUO group was 20.57 ± 19.74% (mean ± standard deviation) lower than that in the MHO group (31.69 ± 18.08%). The sample size was determined via a two-sample *t*-test power calculation with effect size (*d* = 0.596), power of 0.8, and level of significance (α = 0.05). The result indicated that a minimum of 45 subjects per group were needed, and thus, we selected an MUO:MHO ratio of 1:1.6 to increase statistical power for the test.

The authors apologize for this error and state that this does not change the scientific conclusions of the article in any way.

## Conflict of interest statement

The authors declare that the research was conducted in the absence of any commercial or financial relationships that could be construed as a potential conflict of interest.

