# Supplementary material for: Natural Killer Cell Activity and Interleukin-12 in Metabolically Healthy versus Metabolically Unhealthy Overweight Individuals
Source: Front Immunol. 2017 Nov 29;8:1700. doi: 10.3389/fimmu.2017.01700 (PMC5712537; doi:10.3389/fimmu.2017.01700)
Supplement: Supplementary file 1 [file Presentation_1.PDF]

Supplementary Material

Natural killer cell activity and interleukin-12 in metabolically healthy versus metabolically unhealthy overweight individuals

Minjoo Kim,[1] Minkyung Kim,[1] Hye Jin Yoo,[2] Jong Ho Lee[1-3*]

*** Correspondence:** Jong Ho Lee: jhleeb@yonsei.ac.kr

Supplementary Data 1. Detailed method for dietary assessment

The subjects' usual diets were assessed using a semi-quantitative food frequency and 24-h recall method. All subjects were given written and verbal instructions by a dietitian on how to complete a 3-day (2 weekdays and 1 weekend day) dietary record. The energy values and nutrient contents of the dietary intake were calculated using the Computer-Aided Nutritional Analysis Program (CAN-pro 3.0, Korean Nutrition Society, Seoul, Korea).

Supplementary Data 2. Assessment of plasma adiponectin and serum cytokines

Plasma adiponectin concentrations were measured by an enzyme immunoassay (Human Adiponectin ELISA kit, B-Bridge International Inc., Santa Clara, CA, USA). The absorbances of the resulting color reactions were measured at a wavelength of 450 nm using a Wallac Victor2 Multilabel Counter (PerkinElmer Life Sciences, Turku, Finland). The serum level of TNF-α, interleukin (IL)-6, and IL-1β were measured using a Bio-Plex^{TM} Pro Reagent

Kit on a Bio-Plex^{TM} system (Bio-Rad Laboratories, Hercules, CA, USA) according to the manufacturer's instructions. Serum levels of interferon (IFN)-γ were analyzed using an IFN-gamma High-Sensitivity Human ELISA Kit (Covalab, Villeurbanne, France) according to the manufacturer's instructions. Serum levels of IL-12 were measured using a High-Sensitivity Human IL-12 (P70) ELISA kit (Boster Biological Technology, Pleasanton, CA, USA). The absorbance of the reaction mixtures was read at 450 nm using a Victor×5 Multilabel HTS Reader (PerkinElmer, Waltham, MA, USA).

Supplementary Data 3. Detailed procedure for isolations of PBMCs

Whole blood was mixed with the same volume of RPMI 1640 medium (Gibco, Invitrogen Co., Waltham, MA, USA), gently layered on top of a Histopaque-1077 gradient (Sigma-Aldrich, St. Louis, MO, USA), and then centrifuged at 1800 rpm for 20 min at 15°C. After separation, the PBMC layer was isolated, washed twice, and resuspended in RPMI 1640. PBMCs were cultured with RPMI 1640 containing penicillin/streptomycin (Pen/Strep) for NK cell cytotoxicity assays.

Supplementary Data 4. Detailed procedure for determination of NK cell activity

The cytolytic activity of the NK cells was determined using a CytoTox 96 non-radioactive Cytotoxicity Assay Kit (Promega, Madison, WI, USA), which quantitatively measures the release of the stable enzyme lactate dehydrogenase (LDH) upon cell lysis. To assay NK cell cytotoxic activity, PBMCs isolated from each subject were incubated with K-562 cells. Briefly, PBMCs (effector cells, E) were seeded with 2×10^4 K-562 cells (target cells, T) per well at ratios of 10:1, 5:1, 2.5:1, and 1.25:1. The plates were treated at different E:T ratios

(10:1, 5:1, 2.5:1, and 1.25:1) and incubated overnight at 37°C with 5% CO_2, according to the manufacturer's instructions. The supernatant was harvested and transferred to an enzymatic assay plate with substrate mix added to each well, and the reaction was halted with stop solution; the absorbance of the reaction was recorded at 490 nm by a Victor×5 Multilabel Plate Reader (PerkinElmer, Waltham, MA, USA). To compute corrected absorbance values, the following formula was used to obtain percent cytotoxicity for each E:T ratio:

$$\%\ \text{cytotoxicity} = \frac{\text{experimental - effector spontaneous - target spontaneous}}{\text{target maximum - target spontaneous}} \times 100$$
